# SGK1 inhibition attenuated the action potential duration in patient- and genotype-specific re-engineered heart cells with congenital long QT syndrome

**DOI:** 10.1016/j.hroo.2023.02.003

**Published:** 2023-02-16

**Authors:** Maengjo Kim, Saumya Das, David J. Tester, Sabindra Pradhananga, Samantha K. Hamrick, Xiaozhi Gao, Dinesh Srinivasan, Philip T. Sager, Michael J. Ackerman

**Affiliations:** ∗ Division of Heart Rhythm Services, Windland Smith Rice Genetic Heart Rhythm Clinic, Department of Cardiovascular Medicine, Mayo Clinic, Rochester, Minnesota; † Division of Pediatric Cardiology, Department of Pediatric and Adolescent Medicine, Mayo Clinic, Rochester, Minnesota; ‡ Windland Smith Rice Sudden Death Genomics Laboratory, Department of Molecular Pharmacology and Experimental Therapeutics, Mayo Clinic, Rochester, Minnesota; § Thryv Therapeutics Inc, Montreal, Quebec, Canada; || Cardiovascular Research Center, Massachusetts General Hospital, Boston, Massachusetts; ¶ Cardiovascular Research Institute, Stanford University, Palo Alto, California

**Keywords:** Long QT syndrome, Serum and glucocorticoid regulated kinase-1, SGK1, Treatment, Therapeutic, iPSC

## Abstract

**Background:**

Long QT syndrome (LQTS) stems from pathogenic variants in *KCNQ1* (LQT1), *KCNH2* (LQT2), or *SCN5A* (LQT3) and is characterized by action potential duration (APD) prolongation. Inhibition of serum and glucocorticoid regulated kinase-1 (SGK1) is proposed as a novel therapeutic for LQTS.

**Objective:**

The study sought to test the efficacy of novel, selective SGK1 inhibitors in induced pluripotent stem cell–derived cardiomyocyte (iPSC-CM) models of LQTS.

**Methods:**

The mexiletine (MEX)-sensitive SCN5A-P1332L iPSC-CMs were tested initially compared with a CRISPR (clustered regularly interspaced short palindromic repeats)/Cas9 SCN5A-P1332L variant–corrected isogenic control (IC). The SGK1-I1 therapeutic efficacy, compared with MEX, was tested for APD at 90% repolarization (APD90) shortening in SCN5A-P1332L, SCN5A-R1623Q, KCNH2-G604S, and KCNQ1-V254M iPSC-CMs using FluoVolt.

**Results:**

The APD90 was prolonged in SCN5A-P1332L iPSC-CMs compared with its IC (646 ± 7 ms vs 482 ± 23 ms; *P* < .0001). MEX shortened the APD90 to 560 ± 7 ms (52% attenuation, *P* < .0001). SGK1-I1 shortened the APD90 to 518 ± 5 ms (78% attenuation, *P* < .0001) but did not shorten the APD90 in the IC. SGK1-I1 shortened the APD90 of the SCN5A-R1623Q iPSC-CMs (753 ± 8 ms to 475 ± 19 ms compared with 558 ± 19 ms with MEX), the KCNH2-G604S iPSC-CMs (666 ± 10 ms to 574 ± 18 ms vs 538 ± 15 ms after MEX), and the KCNQ1-V254M iPSC-CMs (544 ± 10 ms to 475 ± 11ms; *P* = .0004).

**Conclusions:**

Therapeutically inhibiting SGK1 effectively shortens the APD in human iPSC-CM models of the 3 major LQTS genotypes. These preclinical data support development of SGK1 inhibitors as novel, first-in-class therapy for patients with congenital LQTS.

**Key Findings**

▪ The serum and glucocorticoid regulated kinase (SGK) activity was upregulated 2-fold in the patient-specific induced pluripotent stem cell–derived cardiomyocytes (iPSC-CMs) stemming from the long QT syndrome type 3–causative SCN5A-P1332L pathogenic variant when compared with the patient’s CRISPR (clustered regularly interspaced short palindromic repeats)/Cas9 gene/variant-corrected cell line (isogenic control iPSC-CMs).
▪ Therapeutically inhibiting SGK1 with our novel SGK1 inhibitor effectively shortens the action potential duration (the cellular surrogate for the QT interval) in human iPSC-CM models of all 3 major long QT syndrome genotypes.
▪ As a result of this preclinical data, clinical trials with a novel SGK1 inhibitor in patients with congenital long QT syndrome are anticipated to begin enrolling patients later this year. If this new drug demonstrates appropriate safety and efficacy, it could become the first Food and Drug Administration–approved medication indicated to reduce arrhythmic risk in patients with congenital long QT syndrome.

## Introduction

Long QT syndrome (LQTS) is characterized by delayed repolarization of the myocardium resulting in a prolonged QT interval on the 12-lead electrocardiogram (ECG). Patients with LQTS may present with arrhythmic syncope/seizures, sudden cardiac arrest, or sudden cardiac death (SCD) often following a precipitating event such as exercise, auditory trigger, or extreme emotion.^1^ LQTS occurs in ∼1 in 2000 people.^2^

For patients with LQTS who remain untreated, it is estimated that there is a 50% 10-year mortality in the highest-risk subset.^3^ Current therapeutic options include drug therapy (β-blockers), denervation therapy (left cardiac sympathetic denervation surgery), and/or device therapy (implantable cardioverter-defibrillator). These efforts are usually efficacious.^4^ However, while many patients with LQTS remain protected while on β-blocker therapy, there is a substantial population of LQTS patients experiencing breakthrough cardiac events including implantable cardioverter-defibrillator shocks and SCD.^4^ Furthermore, noncompliance is common with β-blocker therapy due to intolerable side effects.^5^

Approximately 80% of LQTS stems from either loss-of-function (LOF) or gain-of-function (GOF) pathogenic variants in 1 of 3 LQTS-susceptibility genes: *KCNQ1-*encoded I_Ks_ (K_v_7.1) potassium channel (LQTS type 1 [LQT1], ∼35%–40%, LOF), *KCNH2*-encoded I_Kr_ (K_v_11.1) potassium channel (LQTS type 2 [LQT2], ∼30%–35%, LOF), or *SCN5A-*encoded I_Na_ (Na_v_1.5) sodium channel (LQTS type 3 [LQT3], ∼5%–10%, GOF). The LOF or GOF of these critical ion channels underlies the pathological prolongation of the ventricular cardiomyocyte’s action potential duration (APD).^6^

Serum and glucocorticoid regulated kinase-1 (SGK1) is an important regulator of Na_v_1.5-mediated I_Na_ in the heart.^7^^,^^8^ LQT3-causing GOF pathogenic variants in *SCN5A* typically lead to an increase in late I_Na_ current. Small-molecule inhibitors of SGK1 may be antiarrhythmic in cardiac diseases through attenuation of the abnormally increased late I_Na_.^8^^,^^9^ Recently, Bezzerides and colleagues^9^ provided a proof of concept for a SGK1 inhibitor (SGK1-I)–based therapeutic for LQT3 by demonstration of an APD-shortening effect on a patient-specific induced pluripotent stem cell–derived cardiomyocyte (iPSC-CM) model of the LQT3-causing SCN5A-N406K variant following treatment with a novel SGK1-I.^9^

Here, we extend this analysis to test the efficacy of 2 new, potent, and selective SGK1-Is (SGK1-I1 and SGK1-I2) in additional patient-specific iPSC-CM models of LQT3 (SCN5A-P1332L and SCN5A-R1623Q) as well as determine for the first time the potential role of an SGK1-I–based treatment strategy for LQT1 and LQT2.

## Methods

To prevent the reidentification of patients included in this study, individual patient data will not be made available to other researchers. The authors declare that all supporting data are available within the article and its Supplementary Appendix.

### Generation of patient-specific IPSCs

Following written informed consent for this Mayo Clinic Institutional Review Board–approved study (09-006465), iPSCs were generated from peripheral blood mononuclear cells from 4 unrelated patients diagnosed with LQTS; each with a different LQTS-causative pathogenic variant in *KCNQ1* (c.760G>A, p.V254M), *KCNH2* (c.1810G>A, p.G604S) or *SCN5A* (c.3965C>T, p.P1332L and c.4868G>A, p.R1623Q). The peripheral blood mononuclear cells were reprogrammed by Sendai virus transduction using the CytoTune-iPS 2.0 Sendai Reprogramming Kit (Thermo Fisher Scientific, Waltham, MA; A16517) as described previously.^10^ Colonies were picked within 21 days postinfection and clonally expanded for further analysis. CRISPR (clustered regularly interspaced short palindromic repeats)/Cas9 gene-edited/variant-corrected, isogenic control (IC) iPSC lines were engineered by Applied StemCell (Milpitas, CA). All iPSC clones were confirmed to express Tra-1-60, SSEA-3, OCT4, and Nanog pluripotent markers and demonstrated to have a normal karyotype. The presence of the heterozygous pathogenic variant in patient-derived iPSC lines and the genetic correction of the specific variant to wild type in the IC lines were confirmed by Sanger sequencing.

### Cardiomyocyte differentiation

iPSCs were cultured in mTeSR1 media (STEMCELL Technologies, Vancouver, Canada; 85851) in 6-cm dishes precoated with Matrigel (Life Technologies, Carlsbad, CA; A1413302) and incubated at 37°C and 5% CO_2_. At 85% confluence, iPSCs were disaggregated with ReLeSR (STEMCELL Technologies; 05872), passaged into 24-well plates, and allowed to grow for 4 to 5 days to create a monolayer. The differentiation strategy used has been reported previously.^11^ For differentiation, the culture medium was changed to RPMI 1640 GlutaMAX plus 25 mM HEPES supplemented with B27-minus insulin (Life Technologies; A18956-01) containing CHIR99021 (Tocris Bioscience, Bristol, United Kingdom; 4423, 6 μM as working concentration) from days 0 to 2. On day 2, medium was changed to RPMI-B27-minus insulin containing IWP2 (Tocris Bioscience; 3533, 5μM as working concentration) and incubated until day 4. On day 4, the medium was changed back to normal RPMI GlutaMAX-B27-minus insulin and cells were maintained in this media until beating cardiomyocytes appeared, typically around day 6 or day 8. After beating was seen, iPSC-CMs were maintained in RPMI GlutaMAX medium with B27 serum-free supplement (Life Technologies; 17504-044).

### Dissociation of iPSC-CMs

At day 21, iPSC-CMs were washed with phosphate-buffered saline (PBS) (without Ca/Mg) and then subjected to STEMdiff cardiomyocyte dissociation medium (STEMCELL Technologies; 05026). After 5 minutes at 37°C, the iPSC-CMs were pelleted at 300 *g* for 5 minutes and resuspended in plating medium (Dulbecco’s modified Eagle medium, no phenol red, 20% charcoal stripped fetal bovine serum) for subsequent assays. From the following day and onward, the iPSC-CMs were cultured in CM maintenance medium (Dulbecco’s modified Eagle medium, no phenol red, 2% charcoal stripped fetal bovine serum) and media were changed every 2 to 3 days.

### Live cell imaging for APD measurement

iPSC-CMs were cultured on 35-mm glass-bottom dishes (MatTek, Ashland, MA; P35G-1.5-10-C) that were precoated with Matrigel matrix at 37°C, 5% CO_2_. For imaging, cells were incubated at 37°C, 5% CO_2_ for 20 minutes in Tyrode solution containing a volage-sensitive fluorescent dye, FluoVolt, that responds to changes in membrane potential (Thermo Fisher Scientific; F10488). The cells were then washed 3 times in fresh Tyrode solution. During imaging, the dishes were kept in a heated 37°C stage-top environment chamber supplied with 5% CO_2_. Imaging of voltage-indicated cellular APD was taken under a ×40 water objective using a Nikon Eclipse Ti light microscope (Nikon, Tokyo, Japan). Time-lapse videos of multiple, individual beating iPSC-CMs, paced at 1 Hz were recorded at a speed of 20 ms per frame for 20 seconds at 10% LED power. Single regions of interest were selected for every beating iPSC-CM captured in the recordings. The raw data were exported to Excel software (Microsoft, Redmond, WA) and then analyzed with an “in-lab” developed Excel-based program.^11^

### Immunocytochemistry

The iPSC-CMs were fixed with 4% paraformaldehyde for 15 minutes at room temperature (RT) followed by being washed 3 times with PBS. These cells were then permeabilized/blocked with 0.1% Triton X-100/PBS (PBST)/5% goat serum for 45 minutes at RT and subsequently incubated in primary antibody solution made of PBST/5% goat serum containing a 1:250 dilution of Oct4 (Thermo Fisher Scientific; PA5-27438), Nanog (Thermo Fisher Scientific; PA1-097X), Tra-1-60 (Santa Cruz Biotechnology, Dallas, TX; sc-21705), and SSEA-4 (Thermo Fisher Scientific; MA1-021) primary antibodies at RT overnight. The next day, cells were washed 3 times with PBST/5% goat serum at RT before being incubated in PBST/5% goat serum with a 1:250 dilution of Alexa Fluor 488 goat-anti-rabbit (Thermo Fisher Scientific; A-11008), Alexa Fluor 594 goat-anti-mouse (Thermo Fisher Scientific; A-11005), Alexa Fluor 488 goat-anti-mouse (Thermo Fisher Scientific; A-28175), and/or Alexa Fluor 594 goat-anti-rabbit (Thermo Fisher Scientific; A-11012) secondary antibodies at RT for 60 minutes. After secondary antibody incubation, cells were washed 3 times with PBST and finally covered in VECTASHIELD antifade mounting medium with DAPI (Vector Laboratories, Burlingame, CA; H-1300) for imaging. Images were acquired on a Zeiss LSM 780 confocal microscope (Zeiss, Oberkochen, Germany) in the Mayo Microscopy and Flow Cytometry Cell Analysis Core Facility.

### Western blot

iPSC-CMs were lysed in RIPA buffer with Protease Inhibitor Cocktail (Sigma-Aldrich, St Louis, MO; P8340), kept on ice for 20 minutes, and then centrifuged at 14,000 rpm for 15 minutes at 4°C. Supernatant was removed to a new tube. Prior to gel loading, lysate was mixed with 1:1 with 2X loading buffer (Boston BioProducts, Ashland, MA; BP-610), denatured at 95°C for 5 minutes, and then immediately placed on ice. Lysates were aliquoted and kept in –80°C freezer. Proteins were loaded on 4% to 15% TGX gel (Bio-Rad, Hercules, CA; 456-1083) and transferred onto polyvinylidene fluoride membrane using the Trans-Blot Turbo Transfer System (Bio-Rad; 1704150). The membrane was incubated with primary antibody (Phospho-GSK-3β [Ser9] [Cell Signaling Technology, Danvers, MA], 5558S; GSK-3β [Cell Signaling Technology], 9832S; GAPDH [Santa Cruz Biotechnology], sc-32233) diluted in 1:1000 in 5% bovine serum albumin overnight at 4°C. The next day, the membrane was washed with Tris-buffered saline with 0.1% Tween buffer for 45 minutes prior to being incubated in a secondary antibody (goat anti-rabbit [Invitrogen, San Diego, CA], 65-6120; or goat anti-mouse [R&D Systems, Minneapolis, MN], HAF007) diluted 1:10,000 in bovine serum albumin, for 1 hour at RT. The membrane was then washed in Tris-buffered saline with 0.1% Tween for 30 minutes. Finally, the membrane was incubated with SuperSignal West Pico PLUS chemiluminescent ECL substrate (Thermo Fisher Scientific; 34577) for 5 minutes and then exposed to HyBlot CL autoradiography film (Denville Scientific Inc, South Plainfield, NJ; E3012). Film was scanned, and band intensity was analyzed and quantified with ImageJ (Version 1.53e; National Institutes of Health, Bethesda, MD).

### Statistical analysis

All data points are shown as the mean value, and bars represent the SEM. Student’s *t* test was performed to determine statistical significance between 2 groups, and a 1-way analysis of variance and Tukey-Kramer post hoc test were performed for comparisons among 3 or more groups. A *P* < .05 was considered significant.

## Results

The SGK1 activity of the novel SGK1-Is tested was measured in a biochemical assay that assesses the binding and displacement of a wild-type SGK1 active site–directed fluorescent probe and NanoBRET target engagement cell-based assay. The whole-cell SGK1 half maximal inhibitory concentration (IC_50_) of SGK1-I1 and SGK1-I2 was <100 nM. Additionally, their selectivity was assessed in a biochemical assay that assessed the kinase reactivity in a panel of 50 kinases and showed very high selectivity. In addition, there were no effects on cardiac ion channels at <100 times the free tested exposures.

### Generation of patient-specific iPSCs and CRISPR/Cas9–engineered IC iPSCs

Experiments were performed on a mexiletine (MEX)-sensitive SCN5A-P1332L iPSC-CM model that was derived from a 4.5-year-old female patient who presented an ECG rate-corrected QT (QTc) of 583 ms. A CRISPR/Cas9 SCN5A-P1332L variant–corrected IC iPSC-CM was used as a control. The pluripotency of undifferentiated SCN5A-P1332L iPSCs were demonstrated by immunofluorescence staining of pluripotent markers (NANOG, SSEA4, OCT4, and TRA-1-60) (Supplemental Figure 1A). The patient-specific iPSC line had a normal female karyotype (Supplemental Figure 1B). The presence of the heterozygous SCN5A*-*P1332L variant in patient-derived iPSC lines and the genetic correction of SCN5A-P1332L variant to wild type in the IC iPSC line (Supplemental Figure 1C) were confirmed by Sanger sequencing.

Subsequent efficacy studies were performed on a second LQT3-associated patient-derived iPSC-CM model (SCN5A-R1623Q) as well as LQT1 (KCNQ1-V254M)– and LQT2 (KCNH2-G604S)–associated iPSC-CM models. The quality control analysis of the SCN5A-R1623Q iPSCs, including proper pluripotent marker immunofluorescence staining, normal karyotype, and Sanger sequence confirmation of the variant are shown in Supplemental Figure 2. The quality control analysis for KCNQ1-V254M and KCNH2-G604S iPSCs were reported previously.^12^^,^^13^ Patient demographic information is shown in Supplemental Table 1.

### Pathologic prolongation of the action potential duration in 2 LQT3 iPSC-CMs was attenuated markedly by treatment with SGK1-Is

**Figure 1:**
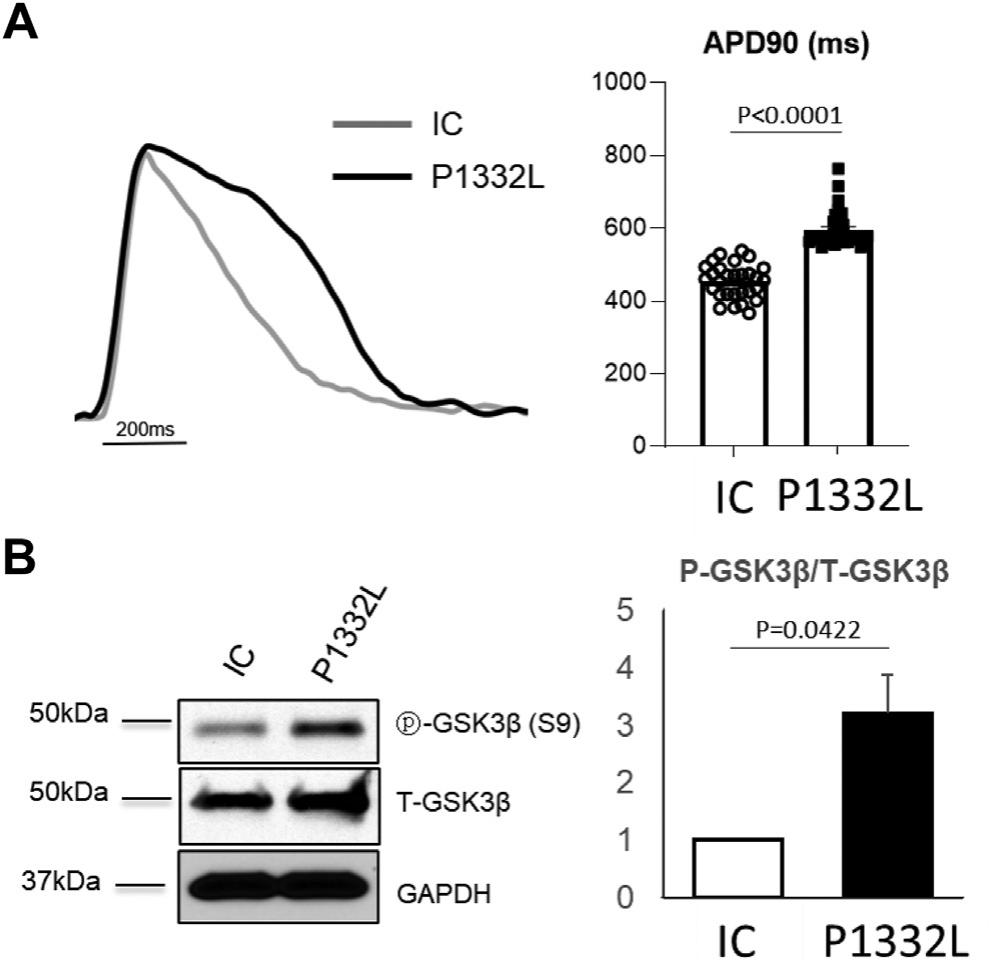
The action potential duration and serum and glucocorticoid regulated kinase-1 activity are increased in SCN5A-P1332L induced pluripotent stem cell–derived cardiomyocytes (iPSC-CMs) compared with isogenic control (IC) iPSC-CMs. **A:** Representative tracings (left) of action potential from SCN5A-P1332L (P1332L) (black line) and IC (gray line) iPSC-CMs. The raw tracings were recorded using a fluorescent voltage dye, FluoVolt. Quantification (right) of action potential duration at 90% repolarization (APD90) of SCN5A-P1332L and IC iPSC-CMs. **B:** Representative immunoblots of protein lysates of SCN5A-P1332L and IC iPSC-CMs probed for phospho (Ser9)-glycogen synthase kinase beta (p-GSK3β), total GSK3β (T-GSK3β), and GAPDH as a loading control (left). Quantification of band intensity on the immunoblot (right). Protein levels were normalized to GAPDH, and data were expressed as fold change relative to IC.

APD of the iPSC-CMs was measured using the voltage-sensitive fluorescent dye FluoVolt. Consistent with the patient’s prolonged QTc (583 ms) on her ECG, the patient-derived SCN5A-P1332L iPSC-CMs displayed a longer APD at 90% repolarization (APD90) (587 ± 9 ms; n = 24; *P* < .0001) (Figure 1A) and a longer APD at 50% repolarization (APD50) (449 ± 4 ms; n = 24; *P* < .0001) compared with the respective IC iPSC-CMs (466 ± 9 ms for APD90 and 281 ± 4 ms for APD50; n = 16) (Figure 1A) when paced at a frequency of 1 Hz.

Interestingly, the SGK activity in the SCN5A-P1332L iPSC-CMs was upregulated by about 2-fold compared with the IC iPSC-CMs, determined by immunoblotting with an antibody against p-GSK3β (phospho [Ser9]-glycogen synthase kinase beta), a well-established SGK1 substrate (Figure 1B).

**Figure 2:**
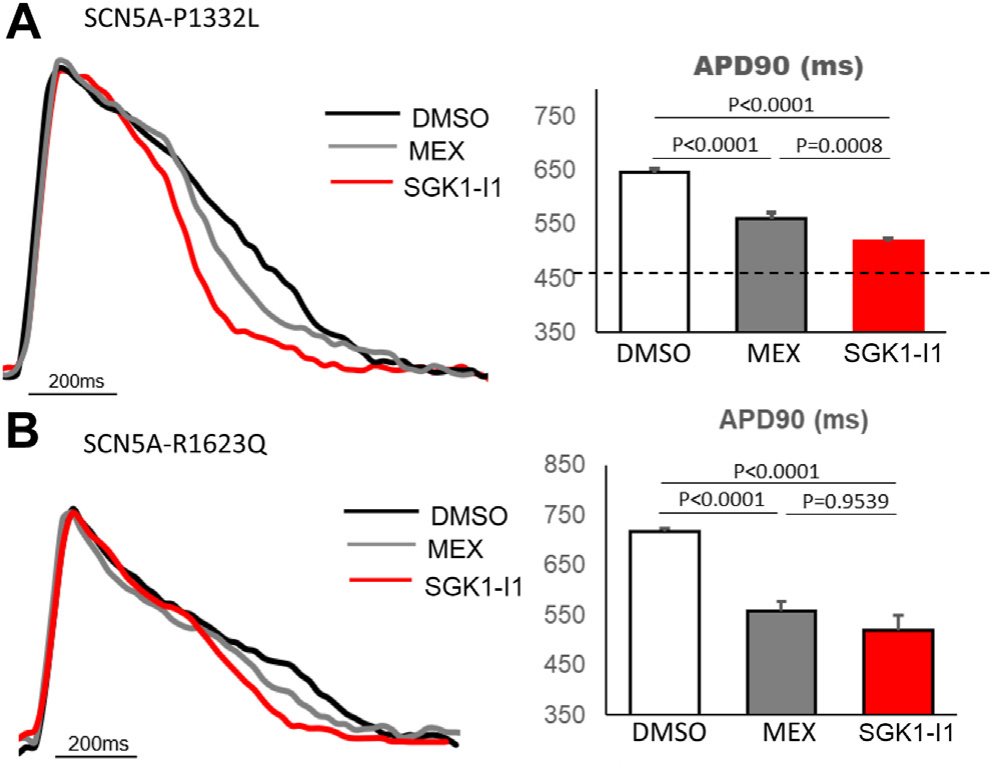
Action potential duration**–**shortening effects of a novel serum and glucocorticoid regulated kinase-1 (SGK1) inhibitor compound in long QT syndrome type 3 induced pluripotent stem cell–derived cardiomyocytes (iPSC-CMs)**. A:** Representative tracings (left) of action potential from SCN5A-P1332L iPSC-CMs after 4-hour treatment with mexiletine (MEX) and a novel SGK1 inhibitor, SGK1-I1. The black, gray, and red lines indicated tracing of action potential of dimethyl sulfoxide (DMSO), 10 μM MEX, and 30 nM SGK1-I1, paced at a frequency of 1 Hz, respectively. Quantification (right) of action potential duration at 90% repolarization (APD90) of SCN5A-P1332L iPSC-CMs after compound treatment. The dotted line indicates the APD90 of isogenic control iPSC-CMs at baseline. **B:** Representative tracings (left) of action potential from SCN5A-R1623Q iPSC-CMs after 4-hour treatment with MEX and SGK1-I1. The black, gray, and red lines indicated tracing of the action potential of DMSO, 10 μM MEX, and 3 nM SGK1-I1, paced at a frequency of 1 Hz, respectively. Quantification (right) of APD90 of SCN5A-R1623Q iPSC-CMs after compound treatment.

The therapeutic efficacy of SGK1-I1 was compared with MEX and with EMD638683 (a commercially available SGK1-I) as described previously.^14^ The APD90 values were recorded 4 hours after treatment using FluoVolt. A dose-response curve for the SGK1-I1 compound at concentrations ranging from 0.03 nM to 3 μM showed a dose-dependent shortening effect of the APD in the SCN5A-P1332L iPSC-CMs, with the greatest effect occurring at a concentration of 30 nM (Supplemental Figure 3A). The APD-shortening effects were observed across a therapeutic window of low concentrations (3 nM to 300 nM) of SGK1-I1; however, at extreme low (0.03 nM) and high concentrations (3 μM) of the compound, the APD-shortening effect was not present. While 10 μM MEX and 5 μM EMD638683 shortened the average APD90 of the SCN5A-P1332L iPSC-CMs from 646 ± 7 ms to 560 ± 7 ms (n = 56; *P* < .0001; 52% attenuation) and to 578 ± 6 ms (n = 52; *P* < .0001; 40% attenuation), respectively, 30 nM SGK1-I1 shortened the APD90 to 518 ± 5 ms (n = 55; *P* < .0001; 78% attenuation) (Figure 2A). Importantly, SGK1-I1 did not shorten the APD in the IC (DMSO APD90: 456 ± 18 ms; n = 18; after treatment with 30 nM of SGK1-I1: 457 ± 20 ms; n = 27; *P* = .8273). A second novel SGK1-I compound (SGK1-I2) also demonstrated greatly reduced APD-shortening effects in the SCN5A-P1332L iPSC-CMs at concentrations ranging from 0.1 to 100 nM (Supplemental Figure 3B).

The APD-shortening effects of SGK1-I1 were then tested in a patient-derived iPSC-CM harboring a different LQT3 pathogenic variant (SCN5A-R1623Q). A dose-response curve for the SGK1-I1 compound at concentrations ranging from 0.03 nM to 3 μM showed a dose-dependent shortening effect of the APD in the SCN5A-R1623Q iPSC-CMs, with the greatest effect occurring at a concentration of 3 nM (Supplemental Figure 3C). At baseline, the SCN5A-R1623Q iPSC-CMs displayed an APD90 of 717 ± 7 ms (n = 31). Addition of 3 nM SGK1-I1 greatly shortened the average APD90 of the SCN5A-R1623Q iPSC-CMs to 519 ± 30 ms (n = 25; *P* < .0001) compared with 558 ± 19 ms (n = 31) with 10 μM MEX and 588 ± 28 ms (n = 25) with 5 μM EMD (Figure 2B, Supplemental Figure 3C).

### Pathologic prolongation of the action potential duration in both LQT1 and LQT2 iPSC-CMs was attenuated by treatment with SGK1-Is

**Figure 3:**
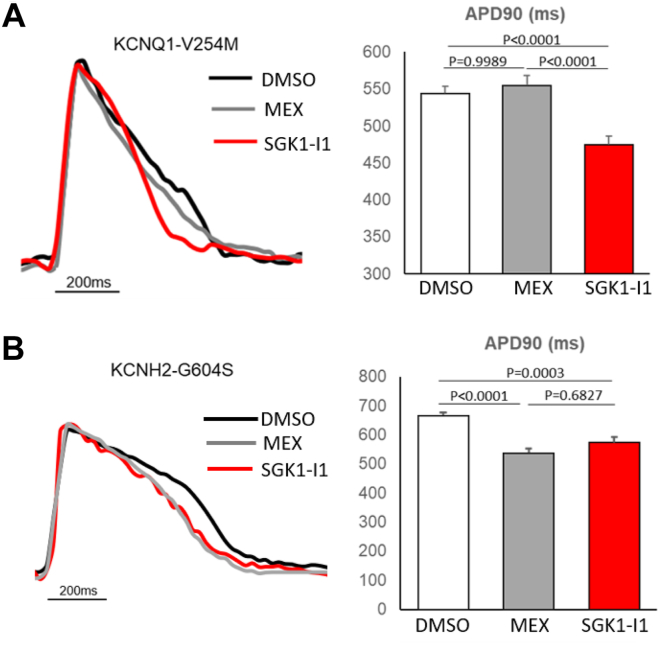
Action potential duration–shortening effects of a novel serum and glucocorticoid regulated kinase-1 (SGK1) inhibitor compound in long QT syndrome type 1 and type 2 induced pluripotent stem cell–derived cardiomyocytes (iPSC-CMs)**. A:** Representative tracings (left) of action potential from KCNQ1-V254M iPSC-CMs after 4-hour treatment with mexiletine (MEX) and an SGK1 inhibitor, SGK1-I1. The black, gray, and red lines indicated tracing of action potential of dimethyl sulfoxide (DMSO), 10 μM MEX, and 100 nM SGK1-I1, paced at a frequency of 1 Hz, respectively. Quantification (right) of action potential duration at 90% repolarization (APD90) of KCNQ1-V254M iPSC-CMs after compound treatment. **B:** Representative tracings (left) of action potential from KCNH2-G604S iPSC-CMs after 4-hour treatment with MEX and SGK1-I1. The black, gray, and red lines indicated tracing of action potential of DMSO, 10 μM MEX, and 30 nM SGK1-I1, paced at a frequency of 1 Hz, respectively. Quantification (right) of APD90 of KCNH2-G604S iPSC-CMs after compound treatment.

The APD-shortening effects of the 2 novel SGK1-Is (SGK1-I1 and SGK1-I2) were examined in patient derived KCNQ1-V254M (LQT1) and KCNH2-G604S (LQT2) iPSC-CMs at various concentrations (Supplemental Figures 4A and 4B). Interestingly, while neither 10 μM MEX nor 5 μM EMD reduced the APD90 in the KCNQ1-V254M iPSC-CMs, administration of 100 nM SGK1-I1 reduced the APD90 from 544 ± 10 ms (n = 35) to 475 ± 11 ms (n = 51; *P* = .0004) (Figure 3A, Supplemental Figure 4A). Treatment with 30 nM of the SGK1-I1 compound had a modest APD90-shortening effect on KCNH2-G604S iPSC-CMs (666 ± 10 ms [n = 50] to 574 ± 18 ms [n = 42]; *P* = .0003) compared with 538 ± 15 ms (n = 44; *P* < .0001) (Figure 3B, Supplemental Figure 4B) after 10 μM MEX. The EMD638683 compound had no effect on the APD90 of the KCNH2-G604S iPSC-CMs (666 ± 12 ms; n = 21; *P* > .9999).

## Discussion

The ubiquitously expressed SGK1 is a PI3K-dependent, serine-threonine kinase that regulates a variety of cellular molecules including ion channels, transporters, enzymes, and signaling molecules.^15^^,^^16^ While SGK1 is expressed in essentially all tissues, it is strictly transcriptionally and post-transcriptionally regulated in response to many agonists and under several pathological conditions including glucocorticoids, mineralocorticoids, serum, inflammatory cytokines, and increases in cytosolic calcium ion concentration.^15^ Notably, SGK1 is activated predominantly under pathological conditions, including hypertension, hypertrophic response, heart failure, and other oxidative and mechanical stressors related to electrical remodeling.^8^^,^^15^^,^^17^

Notably, we demonstrated that SGK1 messenger RNA levels and SGK1 enzymatic activity were increased 2-fold in a human iPSC-CM model of LQT3 compared with its IC model as evident by increased expression of p-GSK3β, a well-known SGK1 substrate. The mechanism responsible for increased SGK1 activity in the iPSC-CMs due to the presence of a pathogenic variant in SCN5A is currently unknown. Whether other pathogenic mutations in LQTS-susceptible genes leading to an arrhythmic substrate and possible primary electrical remodeling at the cellular level in response to functional insult can transcriptionally and post-translationally regulate SGK1 expression and activity are unknown.

Chronic SGK1 activation in cardiomyocytes is associated with altered sodium flux with pathological late sodium current and hyperpolarizing shift of the steady-state inactivation of the *SCN5A*-encoded sodium channel, leading to increase in window current I_Na_, resulting in APD prolongation and increased propensity for afterdepolarization arrhythmic events.^7^^,^^8^ Moreover, mice with constitutively active SGK1 have a prolonged QTc, spontaneous ventricular tachycardia, and SCD.^8^ However, inhibition of SGK1 either by germ-line ablation or dominant-negative inhibition does not lead to a notable pathogenic phenotype under basal conditions and appears to be protective against pathological stress, suggesting that small-molecule inhibitors of SGK1 could be antiarrhythmic in cardiac disease through correction of abnormal I_Na_ and a potential therapeutic for LQTS.^8^^,^^9^

Recently, using a computer-aided drug discovery platform, Bezzerides and colleagues^9^ identified a novel class of SGK1-Is that reduced targeted SGK1 activity in primary cultured cardiomyocytes and reversed the SGK1-induced pathological changes in I_Na_ observed in HEK293 cells coexpressing *SCN5A* and a constitutively active form of SGK1. The lead compound selectively reduced late I_Na_ without a significant decrease in peak I_Na_ in mammalian cardiomyocytes. In a human iPSC-CM model derived from a patient with the LQT3-causing SCN5A-N406K pathogenic variant, their first-generation SGK1-I shortened significantly the pathologically prolonged APD and corrected the abnormal phenotype.^9^ Importantly, the SGK1-I did not shorten the APD of iPSC-CMs derived from a healthy individual.^9^

Here, we provide additional data supporting small molecule–based SGK1-Is as a treatment strategy for LQT3 using novel, potent, and selective SGK1-Is. We first tested the efficacy of the novel SGK1-I1 on a MEX-sensitive SCN5A-P1332L iPSC-CM derived from a patient with LQT3 and its CRISPR/Cas9 SCN5A-P1332L variant–corrected IC iPSC-CM line. Interestingly, while MEX attenuated the pathological APD by 52% in this MEX-sensitive variant line, our novel SGK1-I normalized the pathological APD prolongation almost fully (>75%) in the SCN5A-P1332L iPSC-CM model. Akin to the study by Bezzerides and colleagues,^9^ SGK1-I1 did not further shorten the APD in the IC iPSC-CM. Importantly, we further validated the efficacy of the SGK1-I by demonstrating its APD-shortening effect in a second LQT3-causing pathogenic variant, SCN5A-R1623Q. Again, the SGK1-I APD-shortening effect was significantly greater than what was observed with MEX.

Our novel SGK1-Is also shortened the pathologically prolonged APD in patient-derived iPSC-CM models of the 2 most common forms of LQTS, *KCNQ1*-mediated LQT1 and *KCNH2*-mediated LQT2. Here, while the commercially available SGK1-I EMD638683 failed to shorten the APD, our novel SGK1-Is shortened significantly the APD in both KCNQ1-V254M and KCNH2-G604S iPSC-CM models. Interestingly, Bezzerides and colleagues^9^ showed that SGK1 inhibition by SGK1 Morpholino knockdown or injection with SGK1-dominant negative messenger RNA can rescue the 2:1 atrioventricular block phenotype manifestation of prolonged APD in the zebrafish breakdance mutant (*bkd*^–/–^) that is due to a mutation in the zebrafish homologue of the *KCNH2* gene and recapitulates the human LQT2 phenotype.^9^ Additionally, preincubation of homozygous breakdance mutant zebrafish with their lead SGK1-I compound rescued the 2:1 atrioventricular block in a dose-dependent manner.^9^ While the mechanism is not fully understood, these data suggest that SGK1 inhibition may be potentially therapeutic for the 3 most common LQTS subtypes that collectively account for >80% of LQTS.

Importantly, while we have provided validation of the effectiveness of SGK1 inhibition by small-molecule compounds to shorten the pathological APD in LQT3 iPSC-CM models and have shown for the first time the efficacy of SGK1-I to shorten the APD in patient-specific iPSC-CM models of LQT1 and LQT2, further studies in additional human iPSC-CM models of LQT1, LQT2, and LQT3 with unique pathogenic variants with differing underlying cellular mechanisms of disease are warranted. There may be genotype- and variant-specific effects on APD shortening by SGK1-Is. Preclinical in vivo and ex vivo studies possibly including transgenic animal models will be informative to further advance SGK1-Is as a novel therapeutic strategy for LQTS, though patient studies would be the most definitive.

## Conclusion

Therapeutically inhibiting SGK1 effectively shortened the cardiomyocyte APD in human heart cell models of the 3 major LQTS genotypes. These preclinical data support further development of SGK1-Is as a novel, first-in-class therapy for patients with congenital LQTS.
