# Supplementary material for: SGK1 inhibition attenuated the action potential duration in patient- and genotype-specific re-engineered heart cells with congenital long QT syndrome
Source: Heart Rhythm O2. 2023 Feb 16;4(4):268–74. doi: 10.1016/j.hroo.2023.02.003 (PMC10134391; doi:10.1016/j.hroo.2023.02.003)
Supplement: Supplemental files [file mmc1.docx]

**SUPPLEMENTAL MATERIAL**

**Supplemental Table 1. Summary of patient demographics**

| **Subject** | **Sex** | **Age at Sample Collection (years)** | **Variant** | **Average QTc (ms) [Range] (Sample Size)** | **Signs & Symptoms** | **Treatment** |
| --- | --- | --- | --- | --- | --- | --- |
| 1 | F | 28.1 | KCNQ1-V254M | 512 [486-533] (6) | syncope, QT prolongation, history of near drownings | ICD, former nadolol, former metoprolol |
| 2 | M | 13.1 | KCNH2-G604S | 529 [439-587] (16) | syncope, QT prolongation | mexiletine, nadolol, LCSD |
| 3 | F | 4.5 | SCN5A-P1332L | 486 [433-597] (23) | arrhythmia (in utero), OHCA, VT, QT prolongation | ICD, nadolol, mexiletine, esmolol (former), lidocaine drip (former) |
| 4 | M | 3.1 | SCN5A-R1623Q | 479 [343-664] (50) | in utero arrhythmias TdP, QT prolongation, complete heart block, VT, PVC, ventricular ectopy, gallop, atrial tachycardia, ventricular arrhythmias, atrial ectopy | sotalol (in utero), ICD, RCSD, LCSD, continuous lidocaine, propranolol, mexiletine, former pacemaker, former esmolol, former diltiazem, former magnesium gluconate (for heart?), former flecainide, heart transplant, former milrinone, ablation, ECMO |

F=female, M=male; OHCA=out of hospital cardiac arrest, VT=ventricular tachycardia, TdP=torsades de pointes, PVC=polymorphic ventricular contractions; ICD=implantable cardioverter defibrillator, LCSD=left cardiac sympathetic denervation, RCSD=right cardiac sympathetic denervation


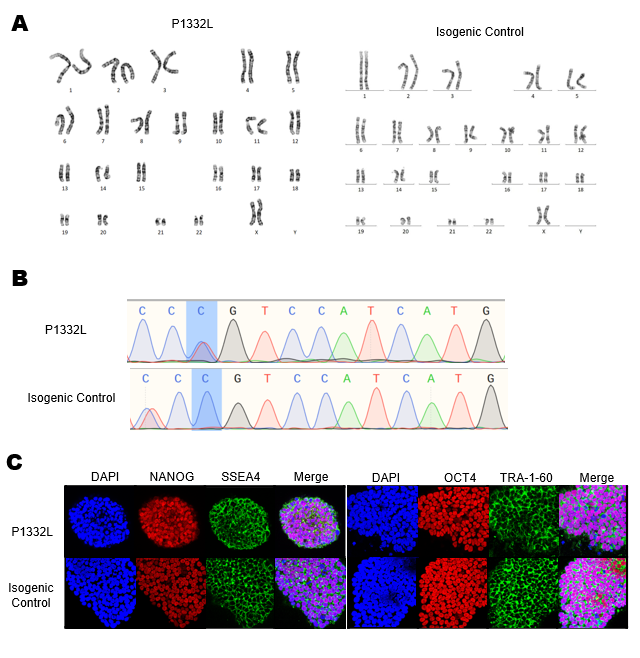


**Supplemental Figure 1. Generation and confirmation of SCN5A-P1332L patient and its gene corrected isogenic control iPS cell lines.** A) The patient (P1332L, left panel) and isogenic “variant corrected” control (right panel) iPSCs showed a normal female karyotype. B) Representative Sanger sequencing chromatograms showing the heterozygous SCN5A-P1332L variant, CCG (P) and CTG(L), in patient iPSCs and wild type sequence, CCG (P) in the isogenic control iPSCs. C) Representative confocal images of induced pluripotent stem cells (iPSCs) reprogrammed from the SCN5A-P1332L positive female patient (top panel) and its gene-corrected isogenic control (bottom panel). Both patient and isogenic control iPSCs demonstrated pluripotent markers (NANOG, SSEA4, OCT4, and TRA-1-60). Scale bar, 50 µm.

**
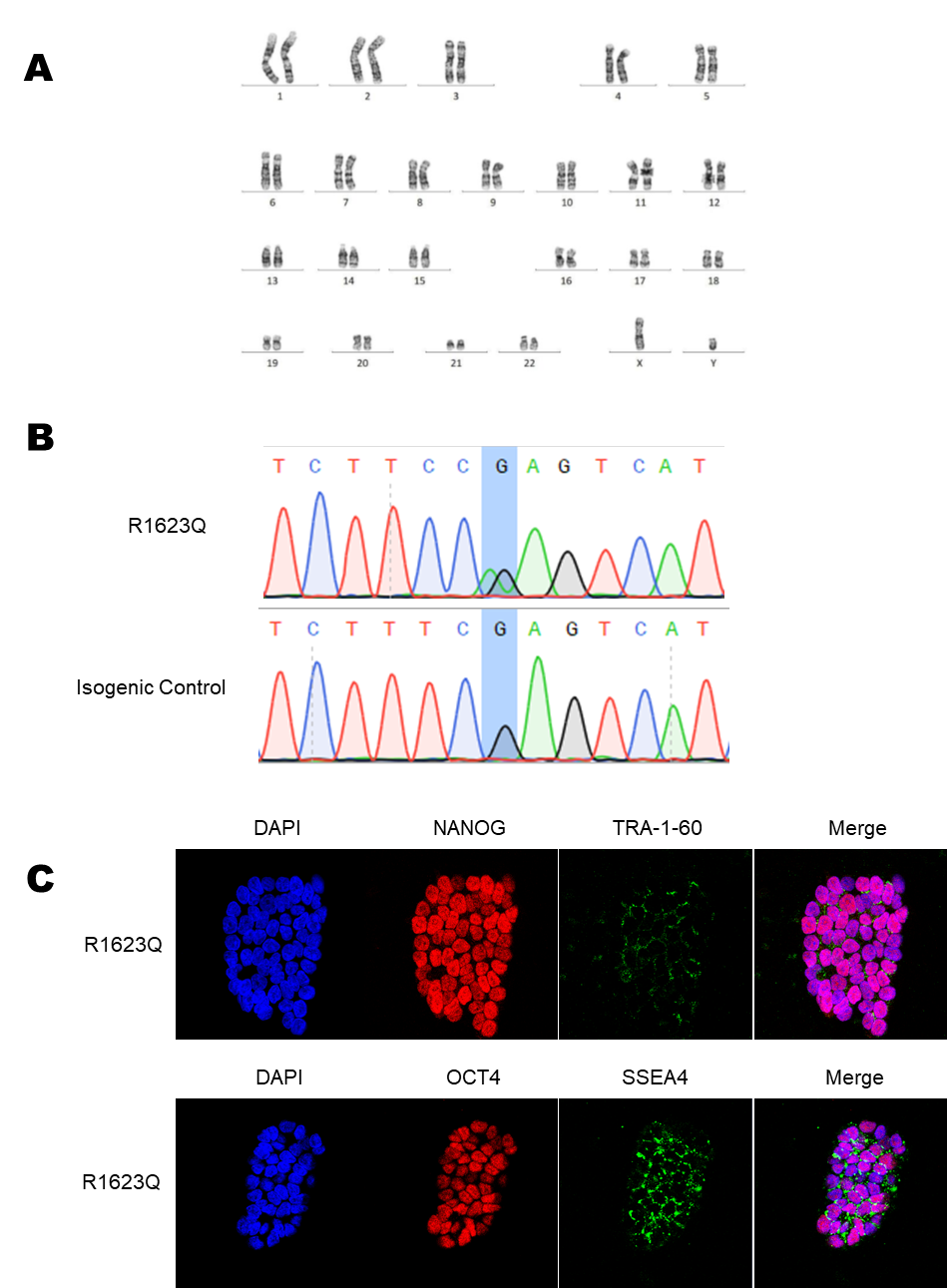
**

**Supplemental Figure 2. Generation of patient-specific SCN5A-R1623Q iPSC-CMs** Shown in panel A is normal female karyotype of the patient-specific iPSCs. Shown in panel B are Sanger sequencing chromatograms confirming the presence of the heterozygous SCN5A*-*R1623Q variant in patient-derived iPSC lines and the genetic correction of SCN5A-R1623Q variant to wild type in the isogenic control iPSC line. Shown in panel A are representative confocal images of undifferentiated patient-specific mutant and isogenic control iPSCs demonstrating four pluripotent markers (NANOG, SSEA4, OCT4, and TRA-1-60).


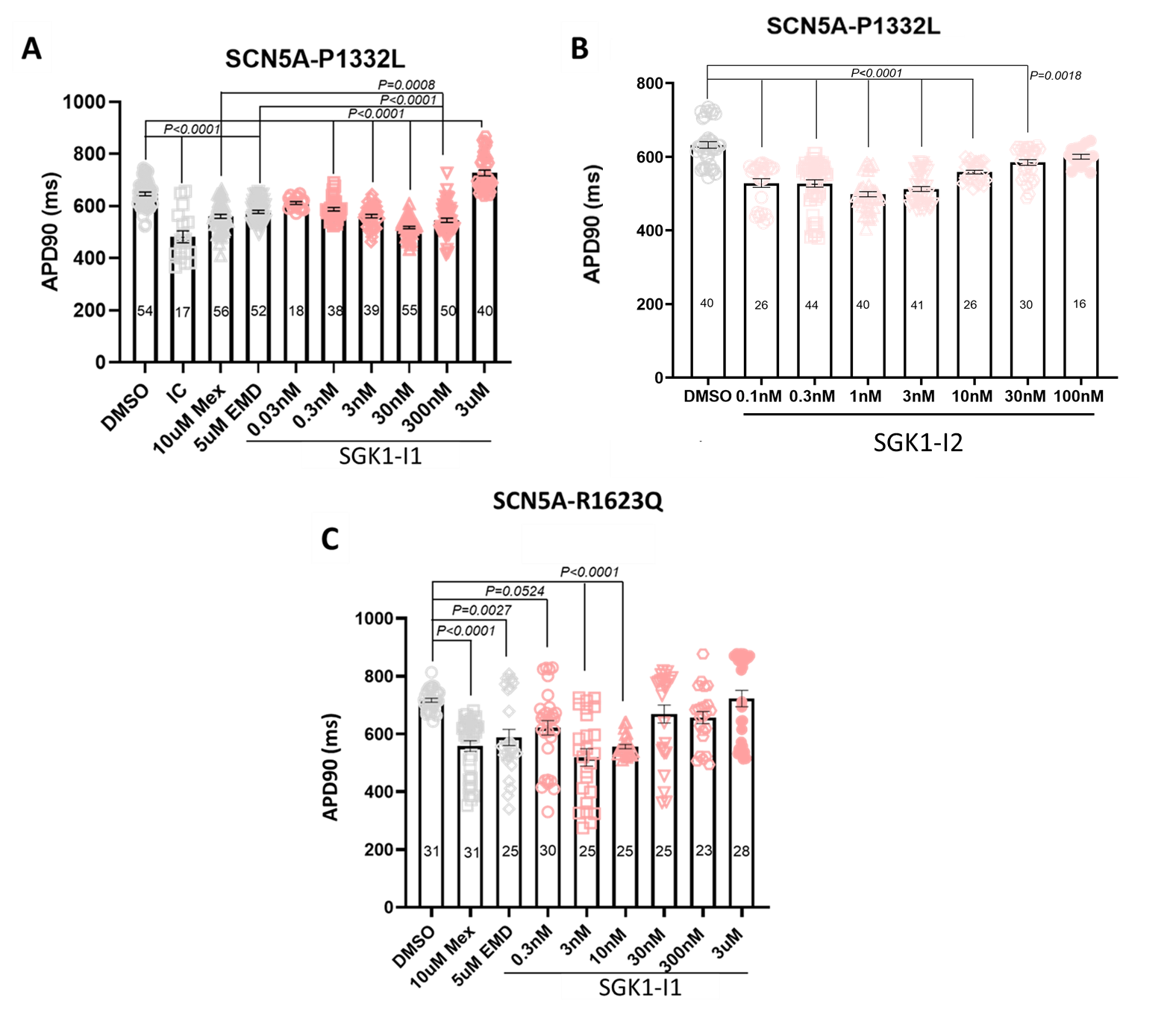


**Supplemental Figure 3.** **Pathologic prolongation of the action potential duration in two LQT3 iPSC-CMs was rescued by treatment with SGK-1 inhibitors in a dose dependent manner.** A) Quantification of APD90 in P1332L-SCN5A iPSC-CMs 4 hours after compound treatment at 0.03nM-3µM SGK1-I1. The data were obtained from 4 independent experiments, and sample numbers (n) referenced in the legend are cumulative. B) Quantification of APD90 in P1332L-SCN5A iPSC-CMs 4 hours after compound treatment at 0.1nM-100nM SGK1-I2. The data were obtained from 4 independent experiments, and sample numbers (n) referenced in the legend are cumulative. C) Quantification of APD90 in R1623Q-SCN5A iPSC-CMs 4 hours after compound treatment at 0.3nM-3µM SGK1-I1. The data were obtained from 3 independent experiments, and sample numbers (n) referenced in the legend are cumulative.

**
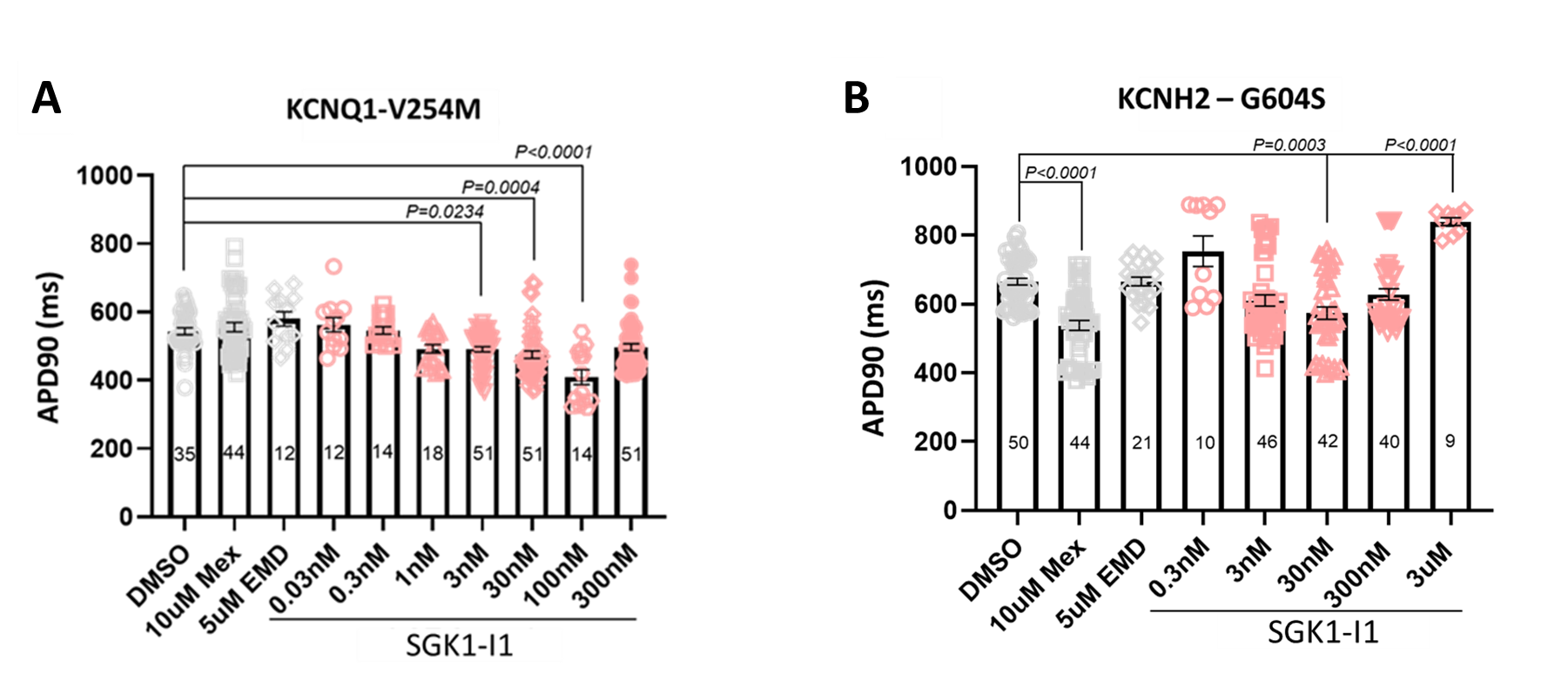
**

**Supplemental Figure 4.** **Pathologic prolongation of the action potential duration in both LQT1 and LQT2 iPSC-CMs was rescued by treatment with SGK-1 inhibitors in a dose dependent manner.** A) Quantification of APD90 in V254M-KCNQ1 iPSC-CMs 4 hours after compound treatment at 0.03nM-300M SGK1-I1. The data were obtained from 4 independent experiments, and sample numbers (n) referenced in the legend are cumulative. B) Quantification of APD90 in G604S-KCNH2 iPSC-CMs 4 hours after compound treatment at 0.3nM-3µM SGK1-I1. The data were obtained from 4 independent experiments, and sample numbers (n) referenced in the legend are cumulative.
